# Clocking in Pillow Time without the Pillow

**DOI:** 10.1371/journal.pbio.0030308

**Published:** 2005-08-23

If you snooze, you lose those uncomely grayish-brown crescents below your eyes. If you don't snooze, you lose a lot more. The body can't fight off infection, the muscles can't regenerate as quickly, the mind can't learn new words, and the eyes can't focus on the road. You also gain things: a bad mood and increased risk for diabetes, high blood pressure, and heart problems. Indeed, the effects of sleep deprivation can be so serious that some sleep scientists liken lifetime sleep debt to a heavy backpack: every sleep hour missed adds an extra pound to your pack until it weighs you down.

For people without time for a daily eight hours in the sack, drugs that counteract the effects of sleep deprivation could serve as substitutes. In a new study, Sam Deadwyler and colleagues have explored this possibility by giving dog-tired rhesus monkeys a drug shown to improve the functioning of alert brains. They found that sleepy monkeys taking the drug performed tasks better and had increased metabolic activity in several regions of their brains. This suggests that the cognitive effects of sleep deprivation can be reduced chemically.

The researchers kept the monkeys awake for 30 to 36 hours by playing music and videos, keeping the lights on, and interacting with them: all the annoyances that can also keep humans from sleeping. To determine the drug's effect on drowsy monkeys, Deadwyler and colleagues used a behavioral test called Match-To-Sample, which measured both accuracy of memory and speed of recall. In the behavioral test, the monkeys saw a simple image flash on a screen. For a variable amount of time, the monkeys had to remember the image. Then, they had to select the correct image from a group of others shown on the monitor simultaneously. When monkeys correctly selected the original image with a cursor, they got a squirt of juice in their mouth as reward. The researchers measured how long they could keep the screen blank between the first and second images without affecting the monkeys' performance. They found that if the monkeys were tired, they couldn't remember the first image for long as they could when they were alert. But with the drug, the sleep-deprived monkeys did at least as well as alert monkeys.

The drug, labeled CX717 (Cortex Pharmaceuticals), acts on AMPA receptors, protein structures on the surface of neurons. When these receptors bind to the neurotransmitter glutamate, they transduce excitatory signals by opening an ion channel. Ampakines including CX717 make the activated channel stay open longer when glutamate binds. More ions pass through the channel, creating a stronger signal when nerve cells are activated. The ubiquity of these receptors makes them good targets for drugs that increase general cognitive functioning. [Fig pbio-0030308-g001]

**Figure pbio-0030308-g001:**
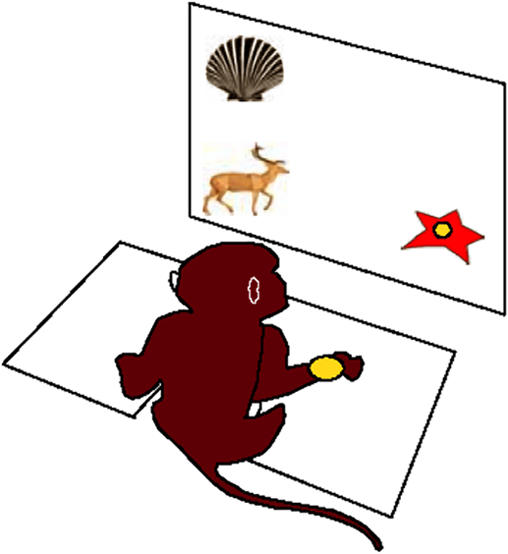
By training monkeys on a classic “match to sample” task, researchers show that ampakine drugs can alleviate the cognitive defects associated with sleep deprivation

The researchers used a technique called positron emission tomography, or PET, to gain insight into CX717's neurobiological role. The PET signal reflected the distribution and rate of metabolism of ingested radioactively labeled glucose in the monkeys' brain cells. By measuring regional brain glucose metabolism, the researchers determined that for sleep-deprived monkeys, glucose metabolism drops off in brain areas previously associated with memory tasks—namely, the prefrontal cortex, the dorsal striatum, and the medial temporal lobe. However, when sleep-deprived monkeys took the drug, they showed heightened glucose metabolism in these same brain regions. The researchers compared these results to suggest a biological basis for the drug's effects.

Previous studies have shown that caffeine and amphetamine can reduce the deleterious cognitive effects of sleep deprivation. But as anybody who has indulged one latte too many knows, caffeine and other powerful stimulants have limited usefulness. These potentially addictive chemicals can distort thinking just as they can enhance it. Because CX717 has a different biochemical action, it may be more beneficial than stimulants for counteracting the cognitive effects of sleep deprivation. But that doesn't mean we should throw away our pillows and blankets just yet: sleep deprivation affects both body and mind.

